# Surgical treatment of awn aspiration causing bronchopleural fistula and bronchiectasis: case reports

**DOI:** 10.1186/s12887-019-1783-1

**Published:** 2019-10-22

**Authors:** Anna Rieth, Tamás Kovács, Zoltán Novák, Katalin Kapus, Aurél Ottlakán, Tibor Németh, József Furák

**Affiliations:** 1 Division of Pediatric Surgery, Department of Pediatrics, University of Szeged, 14-15. Korányi Avenue, Szeged, 6725 Hungary; 2 Division of Pediatric Pulmonology, Department of Pediatrics, University of Szeged, Szeged, Hungary; 3 Division of Thoracic Surgery, Department of Surgery, University of Szeged, Szeged, Hungary

**Keywords:** Awn aspiration, Bronchopleural fistula, Bronchiectasis, Lung resection

## Abstract

**Background:**

Aspiration of grass inflorescences is an extremely rare phenomenon with potential diagnostic difficulties. Due to its special shape, each coughing and respiratory action helps its migration towards the periphery of lung, resulting late-onset, life-threatening complications. The diagnosis has some difficulties for the reason that soon after the aspiration initial symptoms, such as coughing, wheezing or vomiting disappear and bronchoscopy is mostly negative. At least serious complications such as tension pneumothorax, bronchopleurocutaneous fistula or even spontaneous percutan elimination may develope.

**Case presentation:**

We present two cases of pleuropneumonia resulting from aspiration of the head of barley grass. Soon after the accidents initial symptoms diminished, inflammatory markers improved and bronchoscopy was unable to confirm the presence of awn. Despite of conservative treatment (antibiotics, physiotherapy, bronchodilators, expectorants, and inhalation) localized pulmonary inflammation developed after 1 and 9 months showed up on chest computed tomography. After ineffective conservative treatment, surgical resections became inevitable in order to remove chronically inflamed parts (lobectomy, segmentectomy) and foreign bodies. Both patients recovered and were discharged home after successful interventions.

**Conclusions:**

Due to its peculiar shape and behaviour, awn inhalation is a special and atypical form of aspiration, thus great care and awareness is needed in its treatment. Negative bronchoscopic result does not exclude the presence of bronchial grass head. Symptomless child with negative bronchoscopy and improved inflammatory markers should be followed up thoroughly to recognize late complications in time. Regular diagnostic steps (chest ultrasound/X-ray) should be performed to localize potential chronic lung inflammation. Chest computed tomography is a valuable diagnostic tool for identifying and localising the foreign body. In cases with localized inflammation and peripheric localisation, segmentectomy can be a successful and safe alternative of lobectomy.

## Background

Aspiration of foreign body is a common problem especially in case of children, however inhalation of grass head is extremely rare. Grasses (Poaceae) are a family of flowering plants found on fields, roads or along rivers. The well-known barley grass (Horderum morinum) is one of the most widespread species in Europe. Their heads have a special shape with several spikes and bristles, which is responsible for the atypical progression and symptoms of aspiration. After the accident only mild symptoms appear, such as coughing, wheezing or vomiting. The diagnosis has some difficulties for the reason that each coughing and respiratory action helps its migration towards the periphery of the lung, while its transfer to the opposite direction is impossible due to the several spikes. As it gets deeper initial symptoms disappear and bronchoscopy is mostly negative. These negative results and improved physical status may create a false sense of security either in physicians or parents. Nevertheless after a few days aspiration pneumonia, bronchocutaneous fistula, bronchiectasis and other serious even life-threatening complications may develop [1, 2], eventually resulting in surgery [3–5]. Case reports in literature declare that without surgical treatment percutaneous elimination through the chest wall can develop [3, 6–8]. Our cases describe pleuropneumonia and bronchiectasia caused by awn, the bristly head of barley grass.

## Case presentation

### Case 1

A 4-year-old boy was referred to our hospital with a 7-day long history of awn aspiration. On admission, chest X-ray, esophagogram and direct laryngoscopy were performed with negative results. Despite antibiotic therapy (amoxicillin + clavulanic acid) fever developed after 6 days with elevated C-reactive protein (CRP) (38 mg/L), and chest radiography showed atelectasis with pneumonia of the right lower lobe. During bronchoscopy no sign of a foreign body was found, and according to bacteriological culturing of bronchial lavage, antibiotic therapy was completed with netilmycin. On the ninth day of aspiration CRP was 99 mg/L and the intensity of chest pain showed no significant change. Physiotherapy, bronchodilators, expectorants, and inhalation were applied with switching of antibiotic therapy (to trimethoprim/sulfamethoxazole and cefuroxime). One month after the incident the patient was discharged with normal blood work, and chest radiography showing decreased signs of pleuropneumonia. During the next 5 months applied conservative treatment proved ineffective for recurring symptoms. Six months after the foreign body aspiration chest computed tomography (CT) showed bronchiectasis in segments 9 and 10 of the right lung. A second bronchoscopy was carried out, showing purulent secretion with tiny fragments of awn which was removed from the bronchial tree of the right lower lobe. Nine months after the accident, control bronchoscopy revealed signs of inflammation in the right lower bronchi with huge amounts of secretion. Due to recurrent symptoms and confirmation of chronic pneumonia, surgery was decided. After posterolateral thoracotomy, severe adhesions were found between the chest wall and the right lower- and middle lobe, with inflamed hilar lymph nodes. Chronic inflammation and macroscopically abnormal appearance of the whole right lower lobe resulted in lobectomy.

Histopathology confirmed bronchiectasis with chronic inflammation caused by a foreign body blocking the bronchial lumen. After an uneventful postoperative period, the asymptomatic patient was discharged on the 13th postoperative day.

### Case 2

**Fig. 1:**
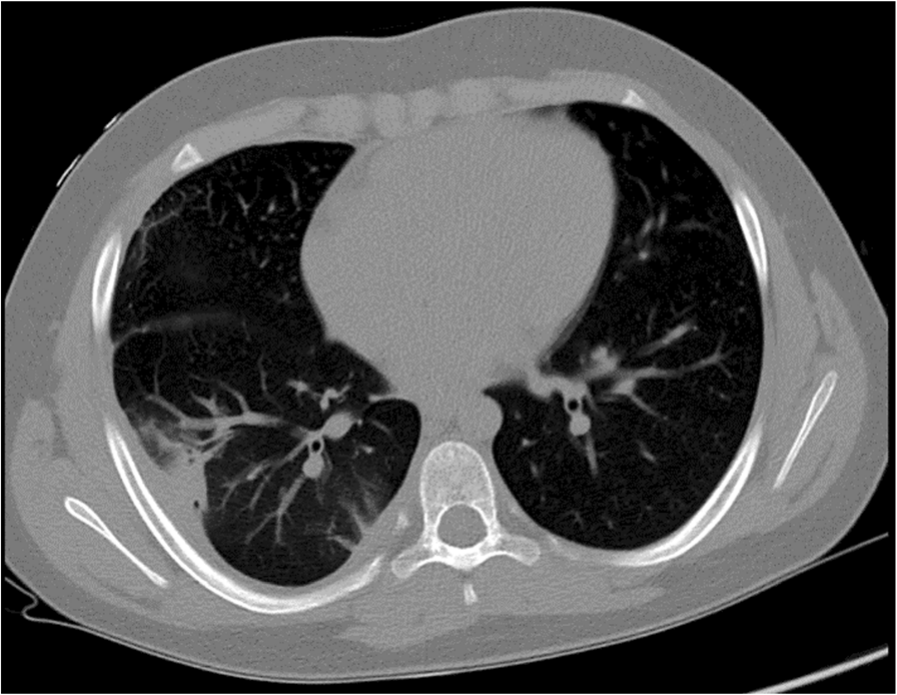
Computed tomography picture of pleuropneumonia in Case 2

A 9-year-old boy presented at our department with a 6-day long history of awn inhalation. At the time of the accident, leading symptoms involved coughing and vomiting. Fragments of awn had also been found in his vomit and stool. After 2 days, fever and chest pain developed, thus chest X-ray was performed in a county hospital showing no apparent abnormalities. Despite having been started on amoxicillin + clavulanic acid and azithromycin, intensity of the right sided chest pain increased. Blood count showed leucocytosis (14,76 × 10^6^/μL) with neutrophil predominance, and elevated CRP (99,4 mg/L). On the sixth day of aspiration repeated chest X-ray revealed right sided infiltration with pleural effusion. Rigid bronchoscopy was performed under general anaesthesia without any endobronchial pathology detected. Bronchial fluid was collected with negative bacteriological results. During an observational period, antibiotic therapy was completed with clindamycin, after which symptoms and blood markers improved on the sixteenth day (white blood cell: 10,000 × 10^6^/μL without left shift, CRP: 19,77 mg/L). Regular chest ultrasound and X-ray confirmed regression of infiltration and effusion, however 16 days later a round shaped lesion with possible abscess formation was detected at the site of the right lower lobe. Chest computed tomography showed a 41 × 20 mm area with decreased air content in segment 6 of the right lung, involving the pleura (Fig. 1).

**Fig. 2:**
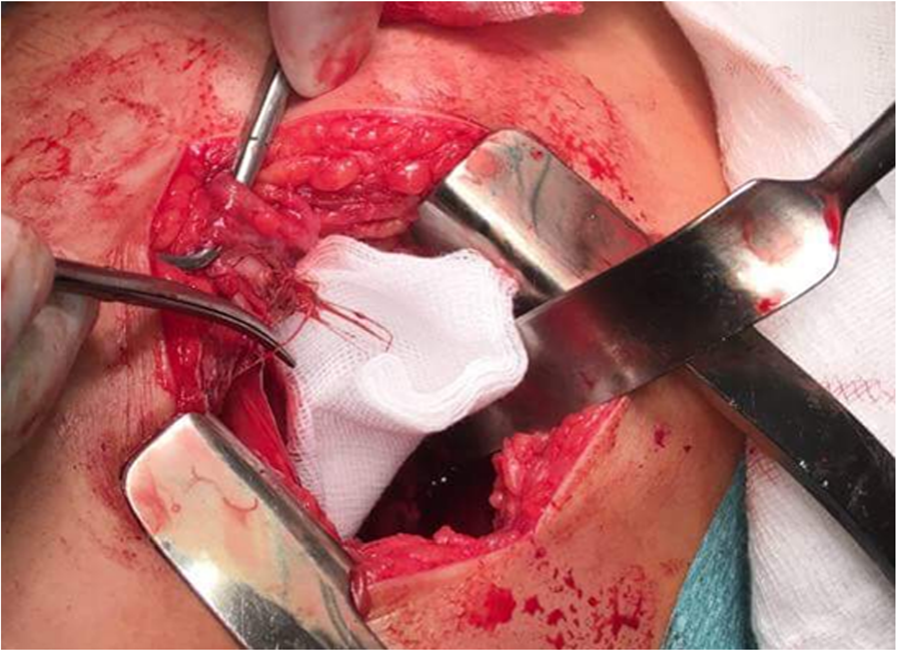
Intraoperative picture of aspirated awn in Case 2

**Fig. 3:**
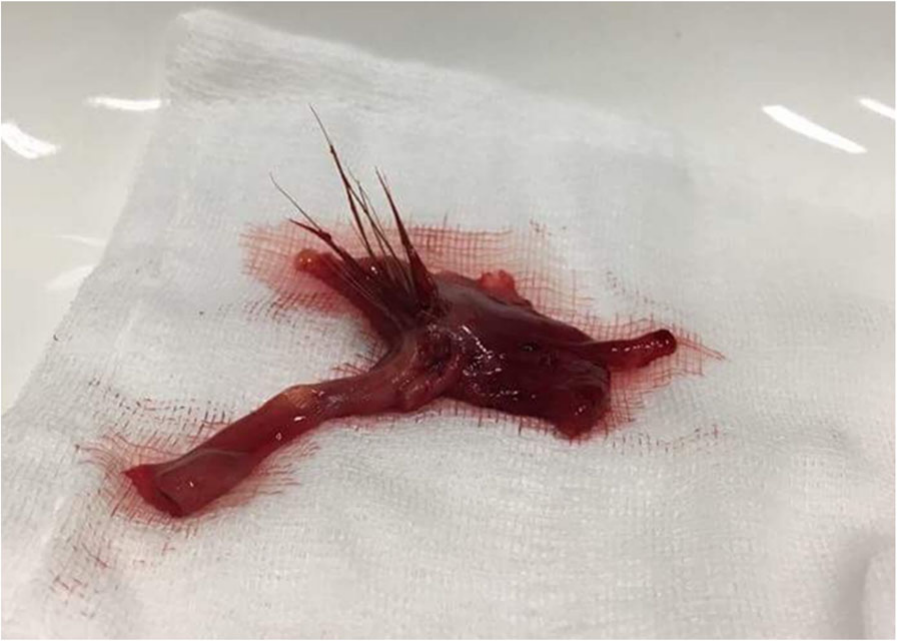
Removed awn specimen in Case 2 with its several spikes, making a unidirectional migration towards the periphery

Surgery was indicated based on the history of awn inhalation, localized lung abscess with pleural effusion, and the known course of awn aspiration, potentially leading to the involvement of intrathoracic organs, and perforation through the chest wall. Focal adhesions between the right lower lobe and the diaphragm were found during posterolateral thoracotomy, and successful removal of a 4-cm-long awn particle from the pleura and the abscess wall around segment 6 was carried out (Figs. 2 and 3). Possible presence of a residual foreign body could not have been excluded, thus surgical resection became inevitable. Segmentectomy was performed due to localized inflammation in segment 6 on CT. Previously described symptoms were relieved after surgery, and follow-up chest radiography showed no signs of the former abscess. The patient was discharged on the 8th postoperative day. Histology confirmed the diagnosis of a chronic granulomatous inflammation resulting from aspiration.

## Discussion and conclusion

Children like to take pieces of toys or food into their mouths and keep them inside while running and playing, so when they fall, they easily aspirate them. This worldwide phenomenon is well- known, however inhalation of grass head is extremely rare. The difference between inhalation of grass inflorescence, or other foreign bodies is mainly due to their shape. A smooth, round object merely overlies the mucosa, however, grass head with its several spikes can migrate towards the periphery. Historically, Chevalier Jackson (1952) was the first to classify grass inflorescences into two different types based on their structure. A small proportion of the grass called the “lodging” type, remains in the respiratory tract and causes pneumonitis. However, the majority, called “extrusive” type travel to the periphery of the lung. Migration continues with each coughing or respiratory action until the grass head finally penetrates through the lung and chest wall, and extrudes spontaneously [1, 6–8]. Immediate bronchoscopy may diagnose some of the “lodging” type heads in a major bronchus [9], but the majority, including the “extrusive” type, rapidly migrate deep beyond the reach of the bronchoscope.

After a few days, as the grass head travels continuously towards the periphery of the lung, initial signs and symptoms may disappear. The following asymptomatic period, with negative bronchoscopy, may create a false sense of security in physicians and parents alike. However, negative bronchoscopy or improved inflammatory markers do not exclude the presence of a bronchial grass head.

Aspiration of grass head is so rare phenomenon, that only few cases were reported in children, and most physicians has limited experience with them. With our two cases we provide diagnostic and management steps to reduce damage and to prevent developing complications.

Reviewing the literature for grass head aspiration in children, the most frequent notated outcomes caused by the grass head migration are chest wall abscess, bronchopleural cutaneous fistula and extrusion of grass head through the chest wall [6–8], pneumothorax [2], pneumomediastinum, recurrent haemoptysis, pleural effusion, bronchiectasis [4], empyema, or osteomyelitis of the rib [1], brain abscess or coexisting acute abdomen [5, 8].

Literary data is consistent, that all the cases with suspicion of a grass head aspiration should immediately be referred for bronchoscopy [1, 3, 6, 9]. However, the removal can only be accomplished before peripheral migration [9]. Due to the anatomical structure of the main bronchi, an aspirated foreign body (including grass heads) is most likely present in the right lower bronchial tree, therefore examination of the right lower lobe during bronchoscopy should be emphasised. According to the revised literature, compared to only 1 left sided case, 15 cases showed inhalation into the right lower lobe [1–9]. Both of our cases showed grass heads in the right lower lobe.

According to the aforementioned rapid migration negative bronchoscopic result do not exclude the presence of bronchial grass head, thus symptomless child with improved inflammatory markers should be followed up thoroughly. Regular chest ultrasound and X-ray is strongly recommended to recognise inflammation and also potential late-onset, life-threatening complications in time. If the examinations show (localising) inflammation computed tomography can be a useful diagnostic tool in identifying the foreign body, and revealing complications. In our cases despite the fact, that both of our patients showed improvement of symptoms, computed tomographic examinations detected bronchiectasis in Case 1 and early stage abscess formation in Case 2, 6 months and 32 days after the aspiration, respectively, suggesting the presence of a foreign body.

Literature provides no exact protocol on “wait-and-see” or surgery. The majority of data state that delayed treatment can be potentially life-endangering and also suggest to perform lobectomy. In order to prevent the above mentioned complications we agree on early surgical exploration of the involved lung. The optimal time for surgical intervention can be determinated by signs of localised inflammation seen on chest X-ray or CT. Despite the fact that pulmonary inflammation may temporarily improve or even vanish, a surgical management should be strongly considered to prevent life-threatening, late complications.

During the first case, 6 months after aspiration, typical features of bronchiectasis were seen on chest X-ray, while in the second case, 32 days after aspiration, an abscess was primarily detected, with successful surgical removal in both cases.

During the first case, due to late diagnosis and extensive pulmonary inflammation, right inferior lobectomy had to be carried out. In the second case, due to localised inflammatory signs, resection of the right 6th segment was satisfactory. According to literature data, lobectomy is most frequently preferred for removal of grass heads [1, 3, 4, 9], with no previous publications of segmentectomy in case of children.

In conclusion we declare that the diagnosis of suspected grass head inhalation may lead to difficulties due to its peculiar shape and behaviour. Negative bronchoscopic result and improved inflammatory markers do not exclude the presence of bronchial grass head, thus symptomless child should be followed up thoroughly to recognise late complications in time. Regular diagnostic steps (such as chest ultrasound or X-ray) should be performed to localise inflammation. Chest CT maybe useful to confirm the diagnosis and identify the position of the foreign body. Surgical removal is opted when the grass head states peripheral, and localised inflammation evolves but before severe complications develop. In case that awn is found in a completely localized inflammation site or abscess, anatomical segmentectomy with the affected bronchus may be the preferred choice. In case of extended inflammation and abscess, or in obscure conditions, lobectomy may be required.

## Data Availability

The datasets used and/or analysed during the current study available from the corresponding author on reasonable request.

## Abbreviations

CRP: C-reactive protein
CT: Computed tomography
